# Guidelines for Parkinson's disease management part II: consensus from the movement disorders scientific department of the Brazilian Academy of Neurology – non-motor symptoms

**DOI:** 10.1055/s-0045-1802962

**Published:** 2025-02-24

**Authors:** Débora Palma Maia, Rubens Gisbert Cury, Pedro Renato P. Brandão, Francisco E. C. Cardoso, Ana Paula Bertholo, André Carvalho Felicio, Arlete Hilbig, Bruno Lopes Santos Lobato, Eline Rozária F. Barbosa, Elizabeth Maria A. B. Quagliato, Gustavo H. C. Sousa, Jacy Bezerra Parmera, Márcia Rúbia, Marcus Vinícius Della Coletta, Maria Sheila Guimarães Rocha, Mariana Spitz, Mônica Haddad, Nina Rosa A. F. Murta, Paulo Caramelli, Raimundo N. D. Rodrigues, Ricardo Nitrini, Roberto Prado, Vitor Tumas, Ylmar Corrêa Neto, Roberta Arb Saba

**Affiliations:** 1 Universidade Federal de Minas Gerais, Hospital das Clínicas, Departamento de Neurologia, Belo Horizonte MG, Brazil.; 2 Universidade de São Paulo, Faculdade de Medicina, Departamento de Neurologia, Centro de Distúrbios do Movimento, São Paulo SP, Brazil.; 3 Hospital Sírio Libanês, Brasília DF, Brazil.; 4 Universidade de Brasília, Hospital da Universidade de Brasília, Brasília DF, Brazil.; 5 Clínica Neurológica, Departamento de Saúde, Congresso Nacional, Brasília DF, Brazil.; 6 Universidade Federal de Minas Gerais, Faculdade de Medicina, Departamento de Clínica Médica/Neurologia, Belo Horizonte MG, Brazil.; 7 Universidade de São Paulo, Hospital das Clínicas, Departamento de Neurologia, São Paulo SP, Brazil.; 8 Hospital Israelita Albert Einstein, São Paulo SP, Brazil.; 9 Hospital São José, Porto Alegre RS, Brazil.; 10 Universidade Federal de Ciências da Saúde de Porto Alegre, Porto Alegre RS, Brazil.; 11 Universidade Federal do Pará, Laboratório de Neuropatologia Experimental, Belém PA, Brazil.; 12 Hospital de Base do Distrito Federal, Brasília DF, Brazil.; 13 Universidade do Estado do Amazonas, Departamento de Neurologia, Escola Superior de Ciências da Saúde, Manaus AM, Brazil.; 14 Hospital Santa Marcelina, São Paulo SP, Brazil.; 15 Universidade do Estado do Rio de Janeiro, Departamento de Neurologia, Rio de Janeiro RJ, Brazil.; 16 Unidade de Sono de Brasília, Brasília DF, Brazil.; 17 Universidade Federal de Sergipe, Faculdade de Medicina, Aracaju SE, Brazil.; 18 Universidade de São Paulo, Faculdade de Medicina de Ribeirão Preto, Departamento de Neurologia, Ribeirão Preto SP, Brazil.; 19 Universidade Federal de Santa Catarina, Faculdade de Medicina, Departamento de Neurologia, Florianópolis SC, Brazil.; 20 Universidade Federal de São Paulo, Departamento de Neurologia, Setor de Transtornos do Movimento, São Paulo SP, Brazil.; 21 Hospital do Servidor Público Estadual de São Paulo, Serviço de Neurologia, Setor de Transtornos do Movimento, São Paulo SP, Brazil.

**Keywords:** Parkinson Disease, Dementia, Autonomic Nervous System Diseases, Pain

## Abstract

The treatment of Parkinson's disease (PD) is a challenge, especially because it is considered highly individualized. The Brazilian Academy of Neurology (ABN) has identified the need to disseminate knowledge about its management, adapting the best evidence to the Brazilian population. The present article aims to report the recommendations for the treatment of non-motor symptoms of PD, developed by a group of specialists in movement disorders from the ABN's scientific department. In 2021, the first part, referring to the motor symptoms of PD, was published. The main non-motor symptoms were addressed—among them neuropsychiatric symptoms, such as depression, anxiety, cognitive alteration, and psychosis—as well as the possible recommended therapies and medications used to control pain, sleep disorders, and dysautonomia.

## INTRODUCTION

Parkinson's disease (PD), described by James Parkinson in 1817, is a neurodegenerative disease characterized by motor symptoms (stiffness, bradykinesia, resting tremor, and postural instability) and non-motor symptoms (neuropsychiatric, sleep, autonomic, and sensory disorders).

The symptoms of PD can be improved through pharmacological, nonpharmacological, and surgical treatments.

The Brazilian Academy of Neurology (ABN) has identified the need to disseminate knowledge about the treatment of PD, adapting the best evidence to Brazilian reality.

In recent years, a group of specialists from the Scientific Department of Movement Disorders of the Brazilian Academy of Neurology has developed a Guide of Recommendations for the Treatment of Parkinson's Disease which has two editions. The constant evolution of therapy and the need to quickly reach the largest number of specialists with updated information led this group to elaborate two articles in the guideline format.

The first part of this guideline deals with the management of motor symptoms (MSs) and was published in *Arquivos de Neuropsiquiatria* in 2022. 1 The second part deals with the treatment of non-motor symptoms (NMSs).

A literature review was performed, and the research sources used were MEDLINE and Cochrane Library, in the period from 1989 to 2024.

To elaborate this guideline, the following topics were searched for in relation to PD:

- Treatment of non-motor symptoms;
- Dementia, mild cognitive impairment, cognition;
- Depression, apathy anxiety, psychosis;
- Dopaminergic dysregulation syndrome;
- Sleep disorders, insomnia, excessive daytime sleepiness, rapid eye movement (REM) sleep behavior disorder, restless legs syndrome, obstructive sleep apnea;
- Pain;
- Autonomic dysfunction in PD, orthostatic hypotension, dysphagia, sialorrhea, urinary dysfunctions, sexual dysfunction.

The classification of studies (4 classes) and levels of evidence (4 levels) were based on the recommendations of the Clinical Practice Guideline Process Manual of the American Academy of Neurology, 2017 edition, 2 as shown in Tables 1 and 2 .

**Table 1:** Classification of evidence for therapeutic studies

Class I	A randomized, controlled clinical trial of the intervention of interest with masked or objective outcome assessment, in a representative population. The **a–e** criteria* is also required.
Class II	A randomized controlled clinical trial of the intervention of interest in a representative population with masked or objective outcome assessment that lacks one criterion **ae** above or a prospective matched cohort study with masked or objective outcome assessment in a representative population that meets **b–e** above.
Class III	All other controlled trials (including well-defined natural history controls or patients serving as own controls) in a representative population, where outcome is independently assessed, or independently derived by objective outcome measurement.
Class IV	Studies not meeting classes I–III criteria, including consensus or expert opinion.

Notes: **a** , concealed allocation; **b** , clearly defined exclusion/inclusion criteria; **c** , clearly defined primary outcome(s); **d** , adequate accounting for dropouts (with at least 80% of enrolled subjects completing the study) and crossovers with numbers sufficiently low to have minimal potential for bias; **e** , for noninferiority or equivalence trials claiming to prove efficacy for one or both drugs, the following are also required: " The authors explicitly state the clinically meaningful difference to be excluded by defining the threshold for equivalence or noninferiority. " The standard treatment used in the study is substantially similar to that used in previous studies establishing efficacy of the standard treatment (e.g., for a drug, the mode of administration, dose and dosage adjustments are similar to those previously shown to be effective). " The inclusion and exclusion criteria for patient selection and the outcomes of patients on the standard treatment are comparable to those of previous studies establishing efficacy of the standard treatment. " The interpretation of the results of the study is based upon a per protocol analysis that takes into account dropouts or crossovers.

**Table 2:** Level of recommendation

A	**Established** as effective, ineffective, or harmful (or established as useful/predictive or not useful/predictive) for the given condition in the specified population.
B	**Probably** effective, ineffective, or harmful (or probably useful/predictive or not useful/predictive) for the given condition in the specified population.
C	**Possibly** effective, ineffective, or harmful (or possibly useful/predictive or not useful/predictive) for the given condition in the specified population.
U	Data inadequate or conflicting; given current knowledge, treatment (test, predictor) is unproven.

## COGNITIVE IMPAIRMENT

Cognitive impairment is a frequent symptom in patients with PD. 3 It can be detected even in the early stages of the disease, usually as cognitive domain-specific deficits or mild cognitive impairment (PD-MCI). 4 Dementia is a typical late complication of the disease, and approximately 80% of patients develop dementia after 20 years of disease. 5 The main risk factors for dementia are advanced age and disease severity, and other important associated factors are the presence of psychosis and a diagnosis of MCI.

Parkinson's disease dementia (PDD) and dementia with Lewy bodies (DLB) are both classified as Lewy body dementias (LBDs) and have similar clinical manifestations and neuropathological substrates. The main difference between them is the chronology of cognitive symptom onset, which is usually late in PDD and very early in DLB. The progression of cognitive symptoms has a significant impact on patients with LBD and compromises the quality of life of the patient, their families, and caregivers. Additionally, it is associated with increased mortality and treatment costs. Therefore, accurate diagnosis and treatment efficiency are essential. 6

In the MEDLINE review, we found 1 study that we classified as class-I, 8 as class-II, 19 as class-III, and 9 as reviews with meta-analysis. We did not classify an additional 18 studies because they did not address the treatment of cognitive symptoms.

### Cholinesterase inhibitors

We found four class-II studies that used donepezil, and one class-III that used rivastigmine. 7 8 9 10 11 Donepezil has shown positive effects on cognitive performance, as assessed by global scales in PD patients with and without dementia. In contrast, a study with rivastigmine showed no efficacy in improving the cognition of patients with PD-MCI. 12 We found no new studies on galantamine. Meta-analysis studies indicated a significant improvement in cognition in patients treated with donepezil (5–10 mg/day) and rivastigmine (6–12 mg/day). 13 14 Cholinesterase inhibitors also caused the overall impression of improvement and reduced neuropsychiatric symptoms. Side effects are uncommon but are more frequent in patients treated with oral rivastigmine. Worsening of parkinsonism or tremors is rarely seen during treatment.

Therefore, donepezil can be useful to treat dementia in PDD (level B). Rivastigmine is possibly useful to treat dementia in PDD (level B). There is insufficient evidence to support or refute the use of galantamine for PDD treatment (level U).

### Memantine

We found one class-II and two class-III studies that used memantine to treat LBD. The analysis of systematic reviews with meta-analysis studies showed that memantine produces a positive clinical impression of change, although it does not promote evident cognitive improvement. 13 15 17 18 19

As such, memantine is possibly effective in treating (PDD) (level B). However, its effectiveness is limited and is restricted to the subjective perception of clinical improvement.

### Other drugs

We found two studies (one class-I and one class-III study) that used rasagiline to treat patients with PD-MCI. 20 21 None of the studies demonstrated the efficacy of the drug in improving patients' cognition.

Thus, we believe rasagiline is not useful in treating cognitive symptoms in patients with PD-MCI (level A).

## GENERAL RECOMMENDATIONS FOR THE PHARMACOLOGICAL TREATMENT OF PDD

Donepezil and rivastigmine are recommended to treat patients with PD-MCI or prodromal DLB.

## DEPRESSION

Depression is one of the main neuropsychiatric manifestations of PD, affecting 40 to 50% of patients. 22

Several classes of medications can be used to treat depression in PD.

### Antidepressants

Venlafaxine and paroxetine were evaluated in a class-I study in PD patients. 23 Treatment effects (relative to placebo), expressed as mean 12-week reductions in the Hamilton depression scale (HAM-D score), were 6.2 points (97.5% confidence interval [CI]: 2.2–10.3, *p* = 0.0007) in the paroxetine group and 4.2 points (97.5% CI: 0.1–8.4, *p* = 0.02) in the venlafaxine XR group. Data on paroxetine are conflicting, as another small trial did not show efficacy. 24 The same class-II small-size trial demonstrated the efficacy of nortriptyline. 24 Another small class-II trial demonstrated the efficacy of both desipramine and citalopram. 25

The evaluation of other antidepressants such as amitriptyline, citalopram, sertraline, and fluoxetine in the treatment of PD depression showed conflicting data. However, as they have established efficacy in depression outside this cohort, they are possibly useful in PD patients. 26

### Dopaminergic agonist

A class-II study evaluating pramipexole for depressive symptoms in patients with PD was conducted by Barone et al. 27 The primary efficacy endpoint, reduced Beck depression inventory (BDI) score, was not met. A secondary endpoint, the BDI responder rate (the proportion of patients with at least a 50% reduction in BDI score from baseline), was met (odds ratio [OR]: 1.8; 95% CI: 1.0–3.1; *p* = 0.05), but it is not clear if the response intensity of symptoms was similar in both groups.

In a meta-analysis published in 2021, pramipexole therapy in PD patients was shown to be effective in controlling depressive symptoms. 28

As such, venlafaxine and pramipexol are likely effective (level B). Paroxetine, desipramine, and nortriptyline are possibly effective (level C). Finally, other antidepressants with proven efficacy outside of PD may be clinically useful (level U).

### Anxiety

Anxiety and anxiety attacks are associated with non-motor fluctuations, especially during off-periods. 29 30 There are no large randomized clinical trials for treating anxiety in PD. 26 A small trial of buspirone showed poor tolerability in this cohort. 31

There is no evidence-based recommendation to manage anxiety in PD. Nonmotor fluctuations must be evaluated (level U).

### Psychosis

Psychosis, characterized by the presence of hallucinations and/or delusions, is frequent in PD and affects approximately 50% of patients at some point during their illness.

All PD patients with psychosis should receive a general medical evaluation and treatment for any precipitating condition, including clinical comorbidities, especially infections and polypharmacy. Regarding antiparkinsonian drugs, the order in which medications should be withdrawn is anticholinergic, amantadine, dopamine agonists, inhibitors of monoamine oxidase B (IMAOB), inhibitors of catechol-O-methyltransferase (ICOMT), and, finally, the reduction of levodopa (L-dopa) when possible. Antidepressants, anxiolytics, and sedatives should be reduced or discontinued. 32 33

The first-generation neuroleptics haloperidol and chlorpromazine have greater affinity for D2-type dopaminergic receptors and are contraindicated in patients with PD. Second-generation antipsychotics (risperidone, olanzapine, quetiapine, clozapine, ziprasidone, and aripiprazole) have a lower affinity for D2 receptors. Despite this, these drugs have a high risk of developing extrapyramidal symptoms and should be avoided by PD patients. 34

### Atypical antipsychotics

Only clozapine and pimavanserin (currently unavailable in Brazil) have class-I positive trials on PD psychosis. 35 36 Regarding clozapine, uncontrolled studies suggest that the drug leads to good control of psychotic symptoms without worsening parkinsonism. 37 38 39 40 41 Placebo-controlled studies have confirmed that low doses of this drug (up to 50 mg/day) are effective in controlling the psychotic symptoms associated with PD. The frequency of the side effects was similar to that of the placebo group. 37 38 42

An important point to consider in patients receiving clozapine is the risk of agranulocytosis. According to the Food and Drug Administration of the United States (FDA), the annual incidence of agranulocytosis with the use of clozapine is of 1.3%. The National Health Surveillance Agency (Agência Nacional de Vigilância Sanitária – ANVISA, in Portuguese) recommends counting white blood cells weekly in the first 6 months. In the following 6 months, it should be done every 2 weeks, then once a month while the treatment lasts.

Regarding quetiapine, uncontrolled studies (class IV) demonstrated improvement in psychosis. 42 43 44 45 46 On the other hand, placebo-controlled class-II studies 47 48 have shown no superiority of quetiapine. One study showed worsening of parkinsonism in 13% of patients. 43 There are two class-II studies comparing quetiapine and clozapine that demonstrate similar efficacy. 49 50 These results should be viewed with caution, as they compare two active drugs without a placebo group. Despite this, quetiapine has been widely used in Brazil and around the world. Furthermore, controlled class-II studies have consistently demonstrated that olanzapine worsens parkinsonian symptoms. 39 51 52 The use of risperidone in the treatment of psychosis in PD has also been associated with worsening parkinsonism in uncontrolled class-IV studies. 53 54

Therefore, clozapine is effective in controlling psychosis in patients with PD (level B). Quetiapine is probably ineffective to control psychosis in PD (level B). Olanzapine is deleterious because of the risk of motor impairment (level B).

### Impulse-control and related behavioral disorders

Impulse-control and related behavioral disorders (ICBDs) occur in up to 20% of the PD population at some point across the disease course and include impulse control disorders (ICD) and dopaminergic dysregulation syndrome (DDS). 55

Impulse control disorders (ICDs) are characterized by pathological gambling, compulsive sexual behavior, binge eating, compulsive shopping, and compulsive hobbyism. 55 56 57 Dopaminergic dysregulation syndrome is characterized by compulsion to ingest L-dopa. During the maximum effect of the medication, manifestations of hypomania may appear with feelings of euphoria, omnipotence, or grandiosity, while its withdrawal induces dysphoria, characterized by sadness, psychomotor slowness, fatigue, or apathy. 56 Both DDS and punding exhibit distinct clinical and pathophysiological characteristics when compared with the other ICDs. 55

There have been no high-quality controlled studies on ICD treatment. A small class-III randomized double-blind crossover study with amantadine 200 mg/day versus placebo showed reduced pathological gambling. 58

In 2024, due to a lack of high-quality evidence, an international expert consensus proposes a definition of severity and treatment pathways for ICBDs in the context of PD. 55 After defining their severity, the recommendation of experts for the treatment of ICD is to gradually taper the dopaminergic agonist (DA) until resolution, or discontinuation of treatment. If the symptoms persist, we suggest reducing L-dopa and/or other dopamine enhancing drugs, such as ICOMTs and IMAOBs. For DDS and punding, the first-line treatment is stopping rescue doses of LD and apomorphine, followed by a reduction in LD. For ICBDs in general, cognitive behavior therapy, quetiapine or clozapine, and antidepressant therapy could be used. In select patients, subthalamic (STN) deep brain stimulation (DBS) should be considered. 55

There are no controlled studies investigating the treatment of ICD or DDS/punding. Stopping rescue doses of LD and apomorphine followed by a reduction in LD is suggested for treatment of DDS/punding (level U). Reduction or suspension of dopaminergic drugs was suggested in the subgroup with ICD (level U).

## SLEEP DISORDERS

Sleep disorders are prevalent comorbidities in PD, affecting a wide range of patients with varying symptoms such as insomnia, excessive daytime sleepiness (EDS), rapid eye movement (REM) sleep behavior disorder (RBD), obstructive sleep apnea (OSA), and restless leg syndrome (RLS).

### Insomnia

Insomnia refers to complaints of difficulty in initiating or maintaining sleep or experiencing early morning awakening. 59 In patients with PD, insomnia requires a personalized approach. Initial interventions should focus on identifying and improving sleep disturbances that may compromise nighttime sleep quality. At the same time, clinicians should assess patients' medication profile for potential culprits, such as antidepressants. Nocturnal motor status is also an important factor, as it can interfere with sleep.

The literature on pharmacological interventions for insomnia in PD presents diverse treatment options but is limited by modest efficacy and methodological shortcomings. Despite its inability to significantly ameliorate sleep parameters, controlled-release L-dopa can mitigate nocturnal akinesia. 60

In a single-center, double-blind, randomized clinical trial involving 93 patients with PD and sleep complaints, trazodone, clonazepam, and melatonin were found to be effective in improving sleep quality, as evidenced by a significant decrease in the Pittsburgh sleep quality index (PSQI) results in all groups. 61 At the same time, trazodone led to a more significant decrease in the Epworth sleepiness scale (ESS) scores. Mild adverse effects have been infrequently reported, underscoring their tolerability.

In a 6-week randomized controlled trial evaluating the efficacy of eszopiclone in PD patients with insomnia, this drug did not significantly extend the total sleep time (TST). Yet, it did improve sleep quality and reduced awakenings compared with the placebo group. 62

In an RCT with moderately-advanced PD patients experiencing sleep problems, rotigotine patches significantly enhanced sleep efficiency, reduced wake after sleep onset (WASO), sleep latency, and boosted REM sleep when compared with placebo. 63 These polysomnography (PSG) improvements correlated with enhanced Parkinson's disease sleep scale (PDSS) and PSQI scores and ameliorated early morning motor symptoms.

In a multinational, double-blind, placebo-controlled trial involving 287 subjects with PD, rotigotine led to significant improvements in early morning motor function and nocturnal sleep disturbances, as assessed by the Unified Parkinson's Disease Rating Scale (UPDRS-III) and PDSS-2 scores, compared with placebo. 64 The most reported adverse events associated with rotigotine are nausea, application-site reactions, and dizziness.

Likewise, melatonin demonstrated a modest yet statistically significant improvement in TST and PDSS in class-II and -III studies. 65 66 In a crossover trial with 40 PD patients experiencing sleep issues, 50 mg of melatonin enhanced objective measures of nighttime sleep duration, whereas 5 mg improved subjective sleep quality and reduced daytime sleepiness. 65

In a single-center, double-blind trial, rasagiline (1 mg/day) did not significantly alter PDSS-2 scores, indicating no perceived improvement in overall sleep quality (level II). 67

Other agents outside the dopaminergic spectrum also offer viable treatment options. Agents such as agomelatine are controversial. Its use is supported only by observational cohort data, for individuals contraindicated for other treatments. 68

As such, rotigotine, trazodone, clonazepam, and melatonin are probably effective in treating insomnia in patients with PD (level B). There is insufficient evidence to support the use of nocturnal extended-release L-dopa, pramipexole, zopiclone, doxepin, rasagiline, and agomelatine (level U). Subcutaneous apomorphine is currently under investigation.

### Excessive daytime sleepiness

The excessive daytime sleepiness (EDS) condition can be conceptualized as an inability to sustain alertness and wakefulness during daytime hours, particularly when circadian drives favor wakefulness. 69 70 71 Managing EDS in patients with PD is a clinical conundrum that requires a tailored approach. Initial interventions should focus on identifying and ameliorating sleep disorders that may compromise nocturnal sleep quality. Concurrently, clinicians should scrutinize patients' medication profile for potential culprits, such as antidepressants, antipsychotics, and sedatives, that might exacerbate hypersomnia. Concerning pharmacological interventions, dopamine agonists (DAs) exhibit a greater risk of inducing EDS than LD, especially at higher doses. 72 73 Moreover, the combination of LD and DAs showed the highest risk of EDS. 74

Studies have shown that modafinil improves patient-perceived wakefulness, but these findings are not corroborated objectively. 75 76 The side effects of modafinil therapy are generally mild and can often be ameliorated with dose adjustment. 77 Methylphenidate has been demonstrated to improve ESS and fatigue scores, but is still investigational, given its limited evidence base and safety concerns. 78 79

A systematic review evaluated the efficacy and safety of modafinil and caffeine in treating EDS in PD. 80 Modafinil showed a statistically significant reduction in EDS as per ESS, with mild adverse events. Caffeine's impact on EDS was nonsignificant in intention-to-treat analysis but significant in per-protocol analysis, with adverse events comparable to placebo, but it is still investigational.

Sleep hygiene education remains a cornerstone, although its efficacy has not yet been empirically quantified (level U). Modafinil is possibly effective in treating EDS in patients with PD (level C). Methylphenidate and caffeine are still under investigation.

### Rapid eye movement sleep behavior disorder

Rapid eye movement sleep behavior disorder is a subclass of parasomnias distinguished by the manifestation of dream-related motor activities resulting from the attenuation of associated muscle atonia. 81

A critical component of the treatment paradigm for RBD involves the comprehensive education of the patient and their bed partner regarding the risk of accidental injuries attributable to dream enactment behaviors. 82 Implementing safety measures in the sleeping environment is highly advisable for mitigating such risks. Additionally, it is incumbent upon clinicians to meticulously review and potentially modify the medication regimen, particularly in the case of pharmaceutical agents known to exacerbate symptoms.

The pharmacotherapeutic landscape for RBD is primarily dominated by clonazepam (CNZ) and melatonin, with the former established by more extensive cohort studies as efficacious at dosages ranging between 0.5 and 2 mg. 82 83 The use of CNZ is effective in treating RBD, benefiting 87 to 90% of patients, although supporting evidence is primarily observational. 82 Despite its effectiveness, this drug carries the risk of side effects, such as sedation, and falls and is contraindicated in moderate-to-severe untreated obstructive sleep apnea syndrome (OSAS). Melatonin serves as an alternative treatment to CNZ for RBD, especially in cases in which CNZ is contraindicated, offering comparable efficacy and a better safety profile. 84 However, both treatments lack high-level evidence, including multicenter, double-blind, placebo-controlled studies with well-defined outcomes.

Various treatments for RBD in PD have been studied, but with limited success and methodological constraints. Sodium oxybate reduced RBD episodes but was not statistically better than placebo. 85 Safinamide showed marked symptom improvement in the majority of participants. 86 However, further studies with the highest number of patients are necessary. Ramelteon seemed to alleviate sleep disturbances in a multicenter open trial but the study design and sample size require cautious interpretation. 87 The use of cannabidiol (CBD) failed to demonstrate a significant impact on RBD frequency. 88 Other pharmacological options, such as pramipexole, rivastigmine, carbamazepine, donepezil, and rotigotine, have been explored but are supported mainly by lower-level evidence, including small sample sizes and open-label designs. 89 90 91 92 93

It is possible that clonazepam and melatonin are effective in treating RBD in patients with PD (level C).

### Restless legs syndrome (RLS)

The RLS constitutes a circadian disorder characterized by disrupted sensory-motor integration, manifesting predominantly as a compulsion to mobilize the legs. 94 This urge is frequently concomitant with, or perceived to be precipitated by, various uncomfortable and disagreeable leg sensations.

Before initiating pharmacological treatment for RLS, an assessment is essential to evaluate the severity, frequency, and duration of symptoms, as well as their impact on patients' quality of life. 95 Mild cases often respond to lifestyle modification. It is also crucial to rule out secondary RLS associated with other medical conditions or deficiencies. 96

In the context of PD, the paucity of controlled studies specifically addressing RLS treatments necessitates that recommendations adhere to those established for the idiopathic (primary) condition. 95 Various pharmacological agents, including dopaminergic agents such as L-dopa, rotigotine, and pramipexole, have demonstrated efficacy in treating idiopathic RLS but require monitoring for augmentation. 97 98 99 100 As alternatives, α2δ ligands, such as pregabalin and gabapentin, including its enacarbil formulation, are efficacious and do not require special monitoring. 101 102 103 104 However, these may induce side effects, such as dizziness and somnolence. In severe treatment-resistant cases, opioids such as oxycodone/naloxone or methadone may be employed, albeit cautiously, due to potential addictive tendencies and sleep-related respiratory issues. 105

A phase II/III clinical trial found that CBD did not significantly alleviate the severity of RLS symptoms in patients with PD when compared with a placebo. 88 106 107 108 Iron supplementation alleviates RLS symptoms in patients with iron deficiency. Other potential interventions, such as intravenous ferric carboxymaltose, exercise, and pneumatic compression devices, are likely efficacious and devoid of special monitoring requirements. 96 109 Nonpharmacological strategies, such as limb massage, thermal baths, or cognitive distraction techniques, may serve as adjunctive measures.

The lack of specific studies for the treatment of RLS in PD does not allow us to make a specific recommendation. Therefore, the recommendation for treatment of RLS outside the context of PD may help the clinician.

### Obstructive sleep apnea (OSA)

It is important to note that OSA can have a significant impact on the quality of life of PD patients, as well as increase the risk of accidents. 110 There is conflicting evidence of this cohort having a higher or lower prevalence than controls. 111

It is essential to consider sleep hygiene measures and the adjustment of antiparkinsonian therapy. 112 Other interventions, such as positional therapy, weight loss, and surgery, may also be effective in selected cases. 113

There are limited treatment options for OSA in patients with PD, but continuous positive airway pressure (CPAP) therapy effectively reduces symptoms and improves quality of life. 110 113 There are at least two randomized controlled trials that investigated the use of CPAP in PD patients with OSA. One study assessed whether this treatment improves cognitive functioning among this cohort. 114 115 Another study found that CPAP improved sleep parameters and daytime sleepiness in patients in this cohort. 116 However, it is essential to note that some patients may have trouble adhering to CPAP therapy.

We believe that CPAP therapy is probably effective in treating OSA in patients with PD (level B).

## FATIGUE

Fatigue is defined as the general feeling of tiredness or difficulty in initiating physical or mental (cognitive) activity, whereas fatigability is the difficulty in maintaining physical or mental activity at a desired level. It is estimated that the prevalence reaches 50%. 117

### IMAOB

A small, low-quality study considered rasagiline possibly useful for the treatment of fatigue in PD. 118

### Nonpharmacological interventions

A negative and low-quality study on patients with PD and fatigue shows insufficient evidence to recommend acupuncture for treatment (level U). 119

Therefore, while rasagiline is possibly useful to treat fatigue in PD patients (level C), there is insufficient evidence to support or refute the use of acupuncture (level U).

## PAIN

Pain in patients with PD is two to three times more frequent than in the population of the same age without it. The estimated prevalence of chronic pain ranges from 30 to 85%. 120 It is important to emphasize that the etiology of pain in this cohort is varied, and its therapeutic approach involves defining the cause.

This symptom is also described as integrating non-motor fluctuations. Therefore, adjusting dopaminergic therapy to reduce fluctuations is the first step in treating pain associated with PD. 121 Different classes of drugs can be useful.

### Duloxetine

Open studies have indicated a positive effect of duloxetine on pain in patients with PD. 122 However, a double-blind, randomized, placebo-controlled trial, class II, failed to confirm the efficacy of duloxetine in treating pain in PD. 123

### Rotigotine

The RECOVER study showed a statistically significant reduction in pain with rotigotine on the Likert pain scale. The doses varied from 2 to 6 mg/24 hours. 64

The post-hoc analysis of this same study demonstrated that rotigotine treatment led to a significant decrease in pain levels compared with placebo in patients with any and in those with moderate-to-severe pain. 124

The DOLORES study was the first double-blind, placebo-controlled study to investigate the effect of rotigotine on pain associated with PD. The primary outcome showed a trend toward improvement in pain intensity but with nonsignificant *p* -values. The sample was small, totaling 60 patients, with 30 receiving rotigotine. 125

### Safinamide

Two main studies, in post-hoc analysis, showed the efficacy and safety of safinamide in the treatment of pain in PD patients. 126 127 These two studies were performed to evaluate the effects of a 100 mg/day dosage on PD patients' pain and motor fluctuation. This analysis showed that the drug significantly reduced the use of pain treatments by approximately 24% and improved specific pain-related items on the PD questionnaire – 39 (PDQ-39). 128

### Opioids

A randomized and controlled study in 47 secondary centers in several European countries in patients with PD and chronic and severe pain, with 202 patients, evaluated the use of the extended-release formulation of oxycodone and naloxone compared with placebo. The primary outcome was based on the mean pain score. The results showed that extended-release oxycodone-naloxone did not significantly reduce the mean 24-hour pain scores after 16 weeks of use. Sensitivity analyses showed a significant difference in the per-protocol population. However, results from exploratory analyses suggest the potential efficacy of an extended-release formulation of oxycodone and naloxone for PD-related pain, particularly specific types of pain, such as severe musculoskeletal and nighttime pain. 129 In a recent study, Brefel-Courbon et al. showed that oxycodone was poorly tolerated in patients with PD compared with L-dopa. Therefore, its use requires caution. 130

The use of opioids in the elderly population requires caution. Therefore, it is necessary to monitor kidney and liver function, observe medication hospitalization, and take measures to reduce side effects. Furthermore, elderly people who have some cognitive impairment must receive medication from a caregiver.

### Anticonvulsants

Pregabalin and gabapentin are drugs used for neuropathic pain and have been frequently administered to patients with PD. 131 There are still no randomized controlled studies regarding these two medications and their results. When used, the patients should be monitored due to the potential risk of worsening motor symptoms. 132

### Acupuncture

A placebo-controlled but nonrandomized study showed acupuncture reduction pain in patients with PD. 133

### Cannabis

There is an ongoing randomized double-blind study evaluating the use of cannabis with fixed percentages of tetrahydrocannabinol (THC) and CBD in pain management. The first phase of the study was published, which evaluated only the highest tolerated dose and safety, without results regarding pain yet. 134

### Physical activity

Observational studies suggest that exercise-induced hypoalgesia may be present in people with PD. Pressure pain thresholds increased in the PD group at all sites tested after all exercise sessions, but with no effect of aerobic exercise dose. 135 There is still a lack of studies using physical activities to treat pain.

Therefore, to treat pain in PD patients, duloxetine is probably not useful (level B), rotigotine is possibly useful (level C), safinamide is probably useful (level B), and oxycodone is possibly useful (level C). There is insufficient evidence to support the use of acupuncture, cannabis, pregabalin and gabapentin, and physical activity (level U).

## CARDIOVASCULAR AUTONOMIC DYSFUNCTION

### Orthostatic hypotension

The main clinical feature of cardiovascular dysautonomia in PD is neurogenic orthostatic hypotension (nOH). It is a common condition (30–60%) that has been associated with an elevated frequency of falls, reduced physical activity, and syncope, even if it is asymptomatic (⅓ of cases).

Management of nOH may be challenging in clinical practice, because many patients also suffer from supine hypertension. Drug use to normalize blood pressure (BP) in the upright position can worsen hypertension when supine.

It is recommended to correct worsening factors before any other intervention and withdraw drugs that reduce intravascular volume (diuretics), induce vasodilatation (sildenafil, nitrates), or block norepinephrine (α blockers, centrally acting α2 agonists, or tricyclic antidepressants). Levodopa and dopamine agonists may also lower BP, so a dose adjustment may be necessary. 135 136

Even though proposed as first line therapy, there is limited low quality clinical evidence for nonpharmacological measures and conclusions are based on a limited number of studies with small sample sizes. The recommendations include higher water intake (∼ 2 L/day), compression stockings, abdominal compression, physical exercise, and physical counter maneuvers. For patients with supine hypertension, resting in a reclining chair during daytime, instead of supine position, is the best treatment. At night, tilting the bed to achieve a 30 or 45° angle with a raised head lowers BP and reduces the exaggerated nocturia and natriuresis, reducing the overnight volume depletion and improving OH in the morning. 137

Most patients require pharmacological therapy to improve symptomatic nOH. The main strategies are expansion of intravascular volume and/or increase of peripheral vascular resistance.

### Fludrocortisone

Fludrocortisone is a synthetic mineralocorticoid that increases renal sodium and water reabsorption, expanding intravascular volume. There is one small class-III study with PD patients showing symptomatic improvement. 26 136

### Midodrine

Midodrine is an oral α1-adrenoceptor agonist that induces vasoconstriction. This drug improves standing BP by increasing both the systolic and diastolic pressures, but carries a risk of significant supine hypertension, so it should not be taken within 5 hours of bedtime. Furthermore, patients should not rest or sleep in the supine position. 26 136 138 This drug is not available in Brazil.

### Droxidopa

Droxidopa is a prodrug that converts into norepinephrine, causing vasoconstriction. Clinically, droxidopa has demonstrated significant symptomatic improvement in nOH, including studies with PD. It is safe and effective for the short-term management of nOH symptoms, but the current evidence is insufficient to confirm its efficacy for long-term. 136 This drug is not available in Brazil.

There is insufficient evidence to recommend the use of pyridostigmine, atomoxetine, domperidone, and fluoxetine in these cases. 137

As such, to treat nOH in PD patients, there is insufficient data to support or refute nonpharmacological treatments (level U), fludrocortisone is possibly effective (level C), midodrine is clinically useful (level A), and droxidopa is clinically useful in the short-term management of this condition (level A).

### Dysphagia

Several swallowing abnormalities are reported in patients with PD. Oral impairment is associated with longer meal duration, fatigue, and reduced swallowing efficiency, which can lead to poor nutritional status and impaired quality of life. 140 Pharyngeal impairment leads to reduced swallowing safety, that is, penetration/aspiration, which is associated with an increased risk of aspiration pneumonia.

Patients with mild-to-moderate dysphagia may benefit from nonpharmacological treatment like postural changes, behavioral changes (e.g., reduced meal volumes and slower eating), and modified meal consistency (e.g., liquid thickeners), besides expiratory muscle strength training and video-assisted swallowing therapy. Expiratory muscle strength training is a behavioral treatment aiming to increase expiratory and submental muscle force production, but there are few studies to confirm this approach. 141

Regarding pharmacological treatment, the role of dopaminergic drugs is controversial, however antiparkinsonian drugs must be optimized. Botulinum toxin injections in the distal esophagus have shown some promise to improve esophageal dysphagia in patients with PD. 140

There is a lack of consensus across studies to determine the effects of DBS on swallowing, likely due to methodological differences. 140

Lee Silverman voice therapy (LSVT) is a standardized and intensive voice training designed specifically for patients with PD. This therapy improved loudness up to two years after treatment as well as speech intelligibility, breath support, and voice quality. The possibility to transfer voice and speech effects of LSVT to swallowing function in patients with PD was investigated by two studies, which yielded low-level evidence that it influences swallowing as a by-product of voice treatment. 141

If dysphagia is severe, avoidance of the oral route by placing a gastrostomy tube should be discussed with the patient. This procedure can ensure adequate nutrition/hydration and possibly reduce the risk of aspiration.

Therefore, there is insufficient data to support standard swallowing therapy (level U), as well as to support or refute LSVT, DBS, and dopaminergic drugs (level U), in the treatment of dysphagia in PD patients.

### Sialorrhea

Sialorrhea, or excessive drooling, occurs not from excessive production of saliva but from the slowing of the swallowing reflex. 142 Behavioral changes (e.g., instructing patients to carefully swallow their saliva at specific times) and radiotherapy were effective for patients with PD in small studies 26 Chewing gum/or sucking on hard candy might provide some relief by stimulating voluntary swallowing. 143

Oral glycopyrrolate (1 mg, twice daily) is efficacious for the very short-term treatment of sialorrhea in PD. Side effects include dry mouth, urinary retention, constipation, and blurry vision. 26

There are class-III studies showing that local administration of anticholinergics (e.g., sublingual atropine drops or ipratropium spray) could be an alternative with no systemic adverse events. 143 Propantheline is another anticholinergic drug used in patients with sialorrhea. However, there are no controlled studies with PD patients. 144

The botulinum toxin A and B (BoNT-A and -B) have been used in the treatment for sialorrhea in PD and have been shown fewer side effects than comparative treatment. 142 Pal et al., showed a marked improvement in the reduction of saliva and a 66% subjective improvement in 9 patients with PD. In 2004, a study by Ondo et al. showed similar efficacy of botulinum toxin B for treatment of drooling in 16 patients with PD.

Special training is needed for performing the injections, and ultrasound guidance may reduce the risk of toxin spreading to nearby anatomical structures. Both the parotid and submandibular glands should be injected to achieve the best effects. 26

In conclusion, to treat sialorrhea in PD patients, there is insufficient data to support behavioral modification and chewing gum (level U). Oral glycopyrrolate is efficacious for the very short-term treatment (level A). Local administration of anticholinergics is possibly clinically useful PD (level C). Both BoNT-A and -B are clinically useful (level B).

### Urinary dysfunction

Dysfunctional urinary symptoms occur in up to 80% of patients with PD, predominantly in men. Irritative urinary symptoms are predominate, usually due to involuntary detrusor muscle contractions (neurogenic bladder) leading to urinary urgency and frequency. 143

A double-blind, randomized, placebo-controlled, 3-site study evaluated the efficacy of the solifenacin succinate in idiopathic PD patients with overactive bladder. The result was negative. 145 However, there were some significant benefits in the active arm, and widespread clinical use was consistent with beneficial results. The most common side effects are a short urinary stream, dry mouth, difficulty in visual accommodation, mental confusion, constipation, and worsening of glaucoma.

The use of mirabegron, a β3-adrenoceptor agonist, for overactive bladder in patients with PD has been studied recently, showing the safety of the drug that can be an option to manage urinary disfunction. However, new studies are recommended to evaluate long-term benefit and safety in this specific population. 146

A small open study evaluated intravesical botulinum toxin type A (BoNT-A) injection in 8 patients with PD and detrusor overactivity refractory to anticholinergics. This injection induced clinical and urodynamic improvement in overactive bladder symptoms that lasted at least 6 months in patients with Parkinson's disease. 147

There is insufficient data to support the use of solifenacin to treat overactive bladder symptoms in patients with PD (level U). To treat urinary incontinence, the use of intravesical injection of TBA is possibly effective (level C).

### Gastrointestinal symptoms

Gastrointestinal symptoms are very frequent in PD. They can precede motor symptoms and have a major impact in quality of life. They include constipation, nausea, early satiety, and bloating. 148

### Constipation

Constipation has a high prevalence in PD, being considered the most common autonomic symptom, and can occur in up to 80% of patients. 149

Lifestyle measures advised for the general population can also help constipation in PD: a fiber-rich diet, bulk laxatives such as psyllium, 149 stool softeners, liquid intake, regular physical exercise, and withdrawal of aggravating factors, such as anticholinergics. 149 In two class-II studies, macrogol, an osmotic laxative that increases the amount of liquids in the bowel, provided good results in PD-associated constipation, by softening the stool. 150 151

In a double-blind, randomized, controlled study, lubiprostone was evaluated as treatment for constipation in PD. This chloride channel activator, which works by enhancing fluid secretion and softening the stools, seemed to be well tolerated and effective for the short-term treatment but there is not enough safety information. 152

A study by Barichella et al., in 2016, offered evidence for the efficacy of fibers combined with probiotics for PD-associated constipation. 153 They are easily available and there are no major side effects. A randomized controlled trial in 2020 investigated the effects of a multi-strain probiotic supplement in PD patients and demonstrated an increase in spontaneous bowel movements in the treated group. 154 155

Abdominal massages are an intuitive method of relieving constipation; however, it was evaluated in a randomized trial with a negative result. 156

Prucalopride, previously described for gastric emptying dysfunction, and lactulose, that was evaluated in a piltot study may be other choices for constipation. 157 158

As such, to treat constipation in PD patients, there is insufficient evidence data to support he lifestyle modifications (level U), macrogol and lubiprostone are possibly useful (level B). Probiotic and prebiotic are clinically useful (level A). There is insufficient evidence data to support or refute abdominal massages (level U).

### Delayed gastric emptying

Delayed gastric emptying (DGE) is very frequent in PD, affecting 70 to 100% of patients. 148 Gastroparesis may contribute to weight loss and malnutrition, in addition to affecting drug absorption. 157 Dopamine replacement itself may contribute to this condition. 148

Dietary modification is advised for all patients, with recommendation of more frequent, smaller meals and avoidance of foods high in fat. 159

A class-IV study showed that domperidone, a prokinetic D2 dopamine receptor antagonist, at a dose of up to 30 mg daily might be useful for treatment of nausea and DGE in PD, since it does not cross the blood-brain barrier in contrast to metoclopramide. 160 161 However, its long-term use may potentially cause adverse cardiac conduction effects. 149

Proton pump inhibitors are frequently used to treat reflux symptoms due to gastroparesis but have been shown to delay gastric emptying and should not be maintained in the long term. 159

Therefore, to treat gastric dysfunction in PD, there is insufficient evidence data to support or refute the use of domperidone (level U).

### Sexual dysfunction (SD)

Often, SD is under recognized in PD. However, it is a frequent nonmotor symptom – present in up to 70% of male and 50% of female patients. The symptoms include decreased libido, erectile dysfunction, premature ejaculation, and orgasm difficulties. This condition is more evident in PD patients with the postural instability-gait disorder subtype. 162 163

There is a paucity of studies evaluating therapeutic interventions for SD, specifically in PD. A class-IV study showed that sex therapy can be useful. 164

Erectile dysfunction is the only SD with available evidence-based treatment. Currently approved treatments focus on drugs that inhibit cGMP-specific phosphodiesterase type 5, such as sildenafil and tadalafil. They increase blood flow in the cavernous bodies of the penis, aiding erection, and its maintenance. Sildenafil is safe and effective in improving erectile functioning in men with PD. 165 The use of these drugs increases the risk of postural hypotension; thus, short-acting medications such as sildenafil may be preferred. 140 Alternatives for erectile dysfunction in PD include intracavernosal injection of alprostadil (prostaglandin E1), vacuum pump devices, and surgical placement of penile prosthesis. 163

Okun et al. showed that transdermal testosterone improved decreased libido and/or erectile dysfunction symptoms in men with PD. 166

Female sexual dysfunction in PD is even less discussed and harder to manage. Women with PD may have inadequate lubrication, loose urine during sex, and have anorgasmia. 167 Therapeutic options are limited and include vaginal lubrication, hormonal therapy, precoital voiding, treatment of overactive bladder, and psychotherapy. 143

Therefore, to treat erectile dysfunction in PD patients, sildenafil is clinically useful (level A).

In conclusion, the treatment of nonmotor symptoms of PD continues to pose a significant challenge for healthcare professionals. Meanwhile, therapeutic options are available, though their effectiveness is variable. Individualized treatments, based on the specific needs of each patient and evidence from the literature, remain essential to optimizing the quality of life for PD patients.
