# Erratum

**DOI:** 10.1055/s-0045-1811960

**Published:** 2025-10-31

In the article “Guidelines for Parkinson's disease management part II: consensus from the movement disorders scientific department of the Brazilian Academy of Neurology – non-motor symptoms”, published in Arq. Neuro-Psiquiatr. 2025;83(1):s00451802962, under DOI number 10.1055/s-0045-1802962 ,

**Where it reads:**

**GENERAL RECOMMENDATIONS FOR THE PHARMACOLOGICAL TREATMENT OF PDD**

Donepezil and rivastigmine are recommended to treat patients with PD-MCI or prodromal DLB.

**Should be:**

**GENERAL RECOMMENDATIONS FOR THE PHARMACOLOGICAL TREATMENT OF PDD**

Donepezil and rivastigmine are **not** recommended for the treatment of patients with PD-MCI or prodromal DLB.

